# Biochemical characterization of a novel exo-oligoxylanase from *Paenibacillus barengoltzii* suitable for monosaccharification from corncobs

**DOI:** 10.1186/s13068-019-1532-6

**Published:** 2019-07-29

**Authors:** Xueqiang Liu, Zhengqiang Jiang, Yu Liu, Xin You, Shaoqing Yang, Qiaojuan Yan

**Affiliations:** 1 Beijing Advanced Innovation Center for Food Nutrition and Human Health, College of Engineering, China Agricultural University, Beijing, 100083 China; 2 College of Food Science & Nutritional Engineering, China Agricultural University, Beijing, 100083 China

**Keywords:** Exo-oligoxylanase, *Paenibacillus barengoltzii*, Characterization, Xylose, Crystal structure

## Abstract

**Background:**

Xylan is the major component of hemicelluloses, which are the second most abundant polysaccharides in nature, accounting for approximately one-third of all renewable organic carbon resources on earth. Efficient degradation of xylan is the prerequisite for biofuel production. Enzymatic degradation has been demonstrated to be more attractive due to low energy consumption and environmental friendliness, when compared with chemical degradation. Exo-xylanases, as a rate-limiting factor, play an important role in the xylose production. It is of great value to identify novel exo-xylanases for efficient bioconversion of xylan in biorefinery industry.

**Results:**

A novel glycoside hydrolase (GH) family 8 reducing-end xylose-releasing exo-oligoxylanase (Rex)-encoding gene (*PbRex8*) was cloned from *Paenibacillus barengoltzii* and heterogeneously expressed in *Escherichia coli*. The deduced amino acid sequence of *PbRex8* shared the highest identity of 74% with a Rex from *Bacillus halodurans.* The recombinant enzyme (PbRex8) was purified and biochemically characterized. The optimal pH and temperature of PbRex8 were 5.5 and 55 °C, respectively. PbRex8 showed prominent activity on xylooligosaccharides (XOSs), and trace activity on xylan. It also exhibited β-1,3-1,4-glucanase and xylobiase activities. The enzyme efficiently converted corncob xylan to xylose coupled with a GH family 10 endo-xylanase, with a xylose yield of 83%. The crystal structure of PbRex8 was resolved at 1.88 Å. Structural comparison suggests that Arg67 can hydrogen-bond to xylose moieties in the -1 subsite, and Asn122 and Arg253 are close to xylose moieties in the -3 subsite, the hypotheses of which were further verified by mutation analysis. In addition, Trp205, Trp132, Tyr372, Tyr277 and Tyr369 in the grove of PbRex8 were found to involve in glucooligosaccharides interactions. This is the first report on a GH family 8 Rex from *P. barengoltzii*.

**Conclusions:**

A novel reducing-end xylose-releasing exo-oligoxylanase suitable for xylose production from corncobs was identified, biochemically characterized and structurally elucidated. The properties of PbRex8 may make it an excellent candidate in biorefinery industries.

**Electronic supplementary material:**

The online version of this article (10.1186/s13068-019-1532-6) contains supplementary material, which is available to authorized users.

## Background

As the huge consumption of fossil fuels results in enormous emission of greenhouse gases, increasing attention has been focused on the consequent air pollution and global warming [1]. To alleviate this problem, biorefinery from renewable lignocellulose biomass tends to be a potential way [2]. Hemicellulose is one of the most abundant sustainable alternatives to petroleum as a platform for generation of biofuels, chemicals and solvents [3]. Xylan is the major component of hemicelluloses, which is composed of β-1,4-linked xylose backbones with various degrees of polymerization and substitution [4]. In biorefinery process, xylans in biomasses should be initially degraded into monosaccharides through chemical, physical or enzymatic methods [5, 6]. Amongst, enzymatic hydrolysis is regarded as a promising strategy as its environmental friendly manner. The complete degradation of xylans requires synergistic reaction of several xylanolytic enzymes, in which xylanases (EC 3.2.1.8) and β-xylosidases (EC 3.2.1.37) play key roles [2]. Generally, endo-xylanases catalyze randomly hydrolysis of the β-1,4 linkages in xylan backbones to produce xylooligosaccharides (XOSs), while β-xylosidases further hydrolyze XOSs to release xylose from the non-reducing ends [7, 8]. To date, several strategies have been performed for xylose bioproduction from xylans of different sources on the basis of co-cation of the two types of xylanolytic enzymes [9, 10]. However, the xylose yields still remain low [11]. Hence, identification of novel xylanolytic enzymes suitable for high-efficient production of xylose from xylan is still of great importance.

So far, a number of xylanases from different sources have been identified and characterized [12]. On the basis of sequence similarities, they have been classified into different glycoside hydrolase (GH) families, including 5, 8, 10, 11, 30 and 43 in CAZy database (http://www.cazy.org). However, the majority of these xylanases are classified into GH families 10 and 11, and only a small group of xylanases fall into GH families 5, 8, 30, and 43 [13]. GH family 8 mainly comprises of endo-xylanases and reducing-end xylose-releasing exo-oligoxylanase (Rex) (EC 3.2.1.156). The later type of enzymes presents a new pattern of action, progressively removing xylose residues from the reducing ends of the substrates (especially XOSs), which is distinct from β-xylosidases releasing xylose from the non-reducing ends [14, 15]. Till now, only 4 Rexs have been identified and biochemically characterized, including the Rexs from *Bacillus halodurans* (BhaRex8) (BAB05824.1) [16], *Bifidobacterium adolescentis* (BaRexA) (AAO67498.1) [17], *Bacteroides intestinalis* (BiRex8A) (EDV05843.1) [18] and *Paenibacillus barcinonensis* (Rex8A) (ALP73600.1) [19]. These Rexs are different from other GH family 8 members in reaction manners and substrate specificities. For example, Rexs catalyze the hydrolysis of the substrates from their reducing ends, and always show high activity on branched XOSs [14, 19, 20]. Though the excellent properties may possess Rexs’ potential in xylan degradation in combination with xylanases, they have a drawback as that they could not catalyze the hydrolysis of xylobiose, the accumulation of which may inhibit the xylanase activity, thus reduce the degradation rate of xylan [16–19]. Therefore, isolation of novel GH family 8 Rexs with xylobiase activity is crucial in enhancing xylan degradation efficiency.

*Paenibacillus barengoltzii* CAU904 was a newly isolated thermophile marine bacterium, which has been reported to produce multiple kinds of enzymes, such as xylanase and chitinase [21, 22]. In this study, we reported biochemical characterization and structure of a novel GH family 8 Rex showing xylobiase activity from *P. barengoltzii* CAU904. The application potential of the enzyme for xylose production from corncob xylan was further evaluated.

## Results

### Gene cloning and sequence analysis of *PbRex8*

**Fig. 1:**
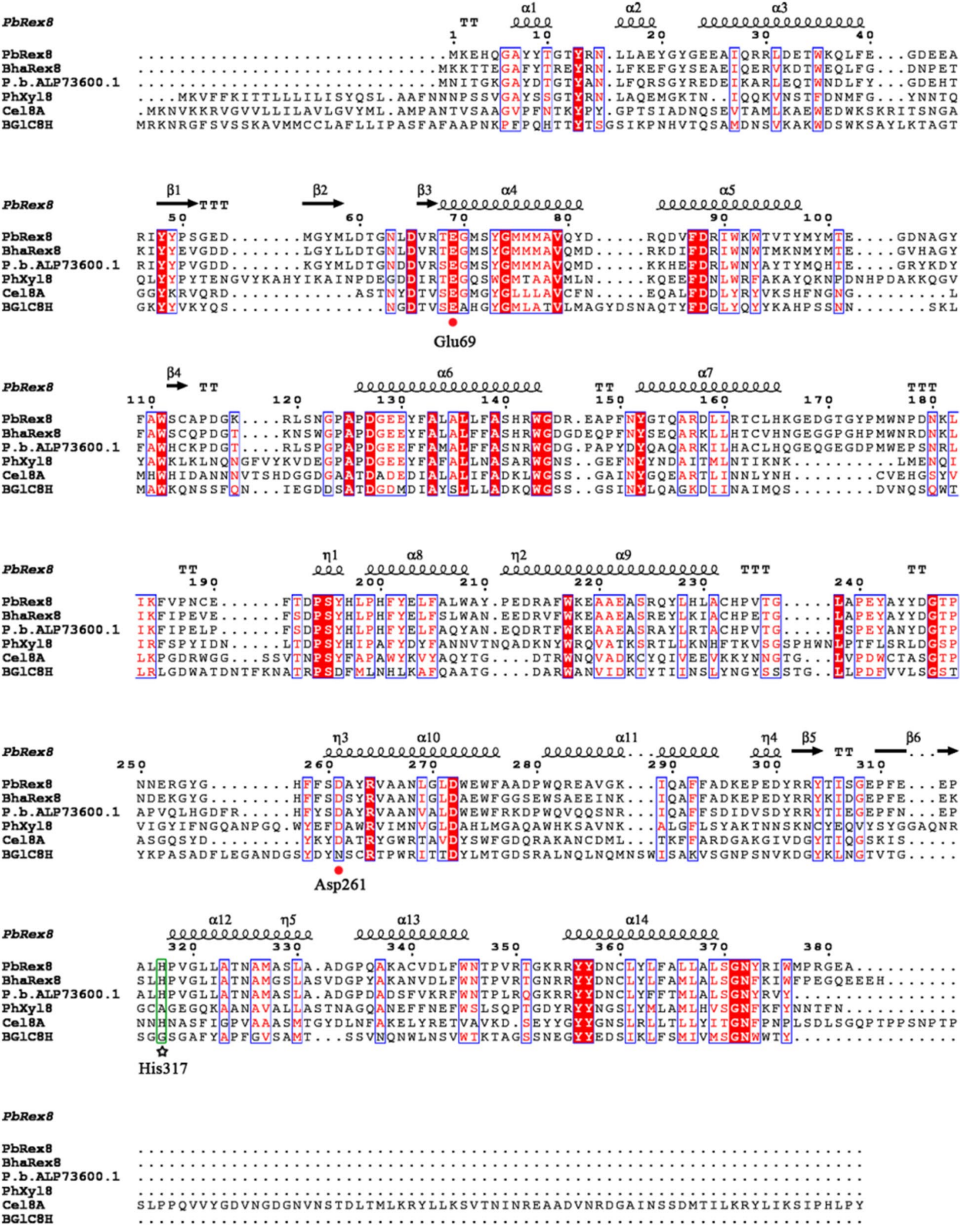
Structural sequence alignment of PbRex8 with other Rexs. Identical residues are shown in white on a red background, and conservative residues are shown in red on a white background. Catalytic residues (Glu69, Asp261) are marked by red dots. Catalytic residue (His317) contributing to recognition of xylose at the reducing end is indicated by an asterisk. Abbreviations of the GH family 8 enzymes in the alignment are as follows: *P. barengoltzii* (PbRex8), *B. halodurans* (BhaRex8), *P. barcinonensis* (*P. b*. ALP73600.1), *P. haloplanktis* (PhXyl8), *C. thermocellum* (Cel8A) and *P*. sp. X4 (BGlC8H)

A reducing-end xylose-releasing exo-oligoxylanase gene (*PbRex8*) from *P. barengoltzii* was amplified. The gene contains an open reading frame (ORF) of 1, 149 bp (Fig. 1), encoding 382 amino acids with a predicted molecular mass of 44 kDa and a theoretical *p*I of 4.8. No signal peptide was predicted in the sequence. The gene sequence has been submitted to NCBI database under accession number KY913838.1.

PbRex8 displayed relatively high identities with the reported GH family 8 Rexs, sharing the highest identity of 74% with the Rex from *B. halodurans* (BAB05824.1) [16], followed by the Rexs from *P. barcinonensis* (ALP73600, 53%) [19], *B. adolescentis* (AAO67498.1, 33%) [17], and *B. intestinalis* (EDV05843.1, 33%) [18] (Fig. 1), suggesting that PbRex8 should be a novel member of GH family 8 Rexs.

### Heterologous expression and purification of PbRex8

**Fig. 2:**
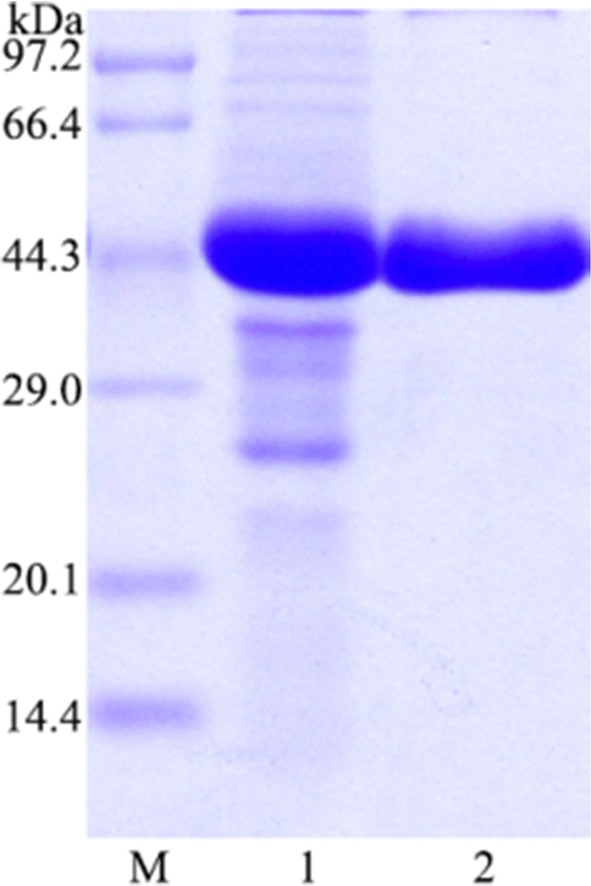
SDS-PAGE analysis of the purified PbRex8. Lane M, low-molecular weight protein standards; lane 1, crude lysate; lane 2, purified PbRex8

The mature protein (PbRex8) without signal peptide was successfully expressed in *E. coli* BL21 (DE3). PbRex8 was purified to apparent homogeneity with specific activity of 331 ± 3 U/mg. The purified enzyme migrated on SDS-PAGE as a single homogeneous band with a molecular mass of 40 ± 2 kDa (Fig. 2), while the native molecular mass was estimated to be 45 ± 3 kDa by gel filtration, indicating that PbRex8 should be a monomer.

### Biochemical properties of PbRex8

**Fig. 3:**
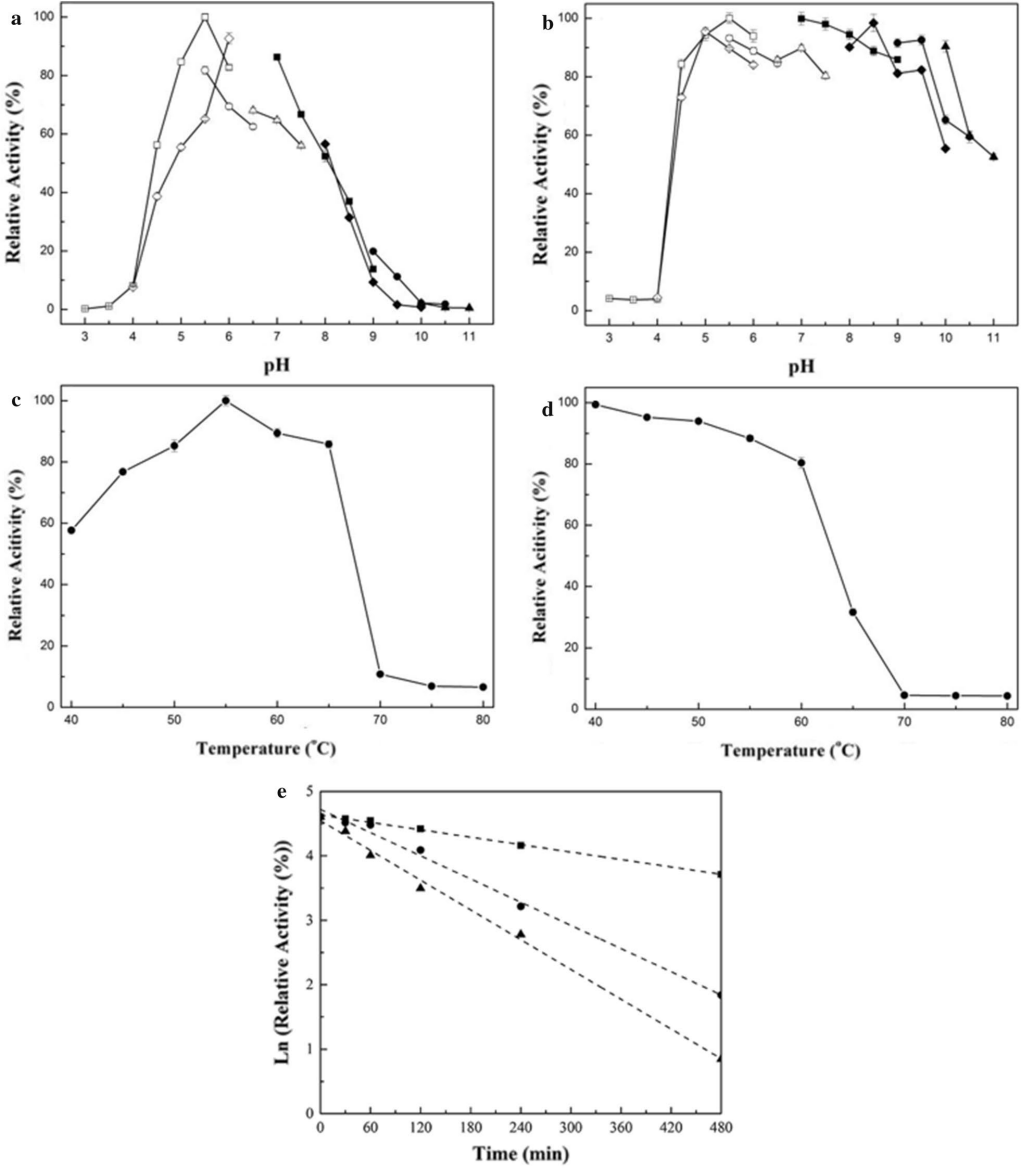
Optimal pH (**a**), pH stability (**b**), optimal temperature (**c**) thermostability (**d**) and thermal denaturation half-lives (**e**) of the purified PbRex8. Symbols for optimal pH and pH stability: citrate (open square, pH 3.0–6.0); acetate buffer (open diamond, pH 4.0–6.0); MES (open circle, pH 5.5–6.5); MOPS (open triangle, 6.5–7.5); Tris–HCl (filled square, pH 7.0–9.0); CHES (filled diamond, pH 8.0–10.0); Gly-NaOH (filled circle, pH 9.0–10.5); CAPS (filled triangle, 10.0–11.0). Thermal denaturation half-lives of PbRex8 were determined at 50 °C (filled square), 55 °C (filled circle) and 60 °C (filled triangle) for 8 h. The values are the average of experiments performed in triplicate

The optimal pH of PbRex8 was found to be pH 5.5 (Fig. 3a), and it was stable in a broad pH range of 4.5–9.5 (Fig. 3b). PbRex8 displayed maximal activity at 55 °C (Fig. 3c), and retained more than 80% of its initial activity after incubation at 60 °C for 30 min (Fig. 3d). The thermal denaturing half-lives of PbRex8 at 50, 55 and 60 °C were 380 ± 15, 135 ± 8 and 82 ± 6 min, respectively (Fig. 3e). SDS (5.3% ± 0.6) strongly inhibited the enzyme’s activity, while the other tested metal ions and reagents exhibited slight or no effects (data not shown).

### Substrate specificity and kinetic parameters of PbRex8

PbRex8 showed relatively high activity towards XOSs (DP 2–6), with the highest activity towards xylotetraose (X4, 343 ± 5 U/mg), followed by xylotriose (X3, 331 ± 3 U/mg), xylopentaose (X5, 237 ± 9 U/mg), xylohexaose (X6, 177 ± 12 U/mg) and xylobiose (X2, 67 ± 1 U/mg) (Table 1, Additional file 1: Fig. S1). Besides, it displayed low activity towards xylans (birchwood xylan, beechwood xylan, oat spelt xylan), β-glucans (barely and oat) and lichenin (Table 1), but no activity towards other substrates tested, including reduced xylotriose, reduced xylotetraose, *p*NP-β-xylopyranoside, locust bean gum, CMC, colloidal chitin and chitosan.

**Table 1:** Substrate specificity of PbRex8 from *P. barengoltzii* CAU904

Substrate	Specific activity (U/mg)^a^	Relative activity (100%)
X_2_	67 ± 1	19.5
X_3_	331 ± 3	96.5
X_4_	343 ± 5	100
X_5_	237 ± 9	69.1
X_6_	177 ± 12	51.6
Birchwood xylan	2.2^c^	–^b^
Beechwood xylan	2.6^c^	–
Oat spelt xylan	1.3^c^	–
Oat β-glucan	1.3^c^	–
Barely β-glucan	1.0^c^	–
Lichenin	0.5^c^	–

All data are mean values ± standard deviations of triplicate measurements

^a^The specific activity of PbRex8 was determined by measuring the enzyme’s activity in citrate buffer (pH 5.5) at 55 °C using various substrates. No enzyme activity was detected towards X3r, X4r, colloidal chitin, CMC, chitosan, locust bean gum and *p*NP-X

^b^Relative activity < 1%

^c^The standard deviations < 0.1

### Hydrolysis properties of PbRex8

**Fig. 4:**
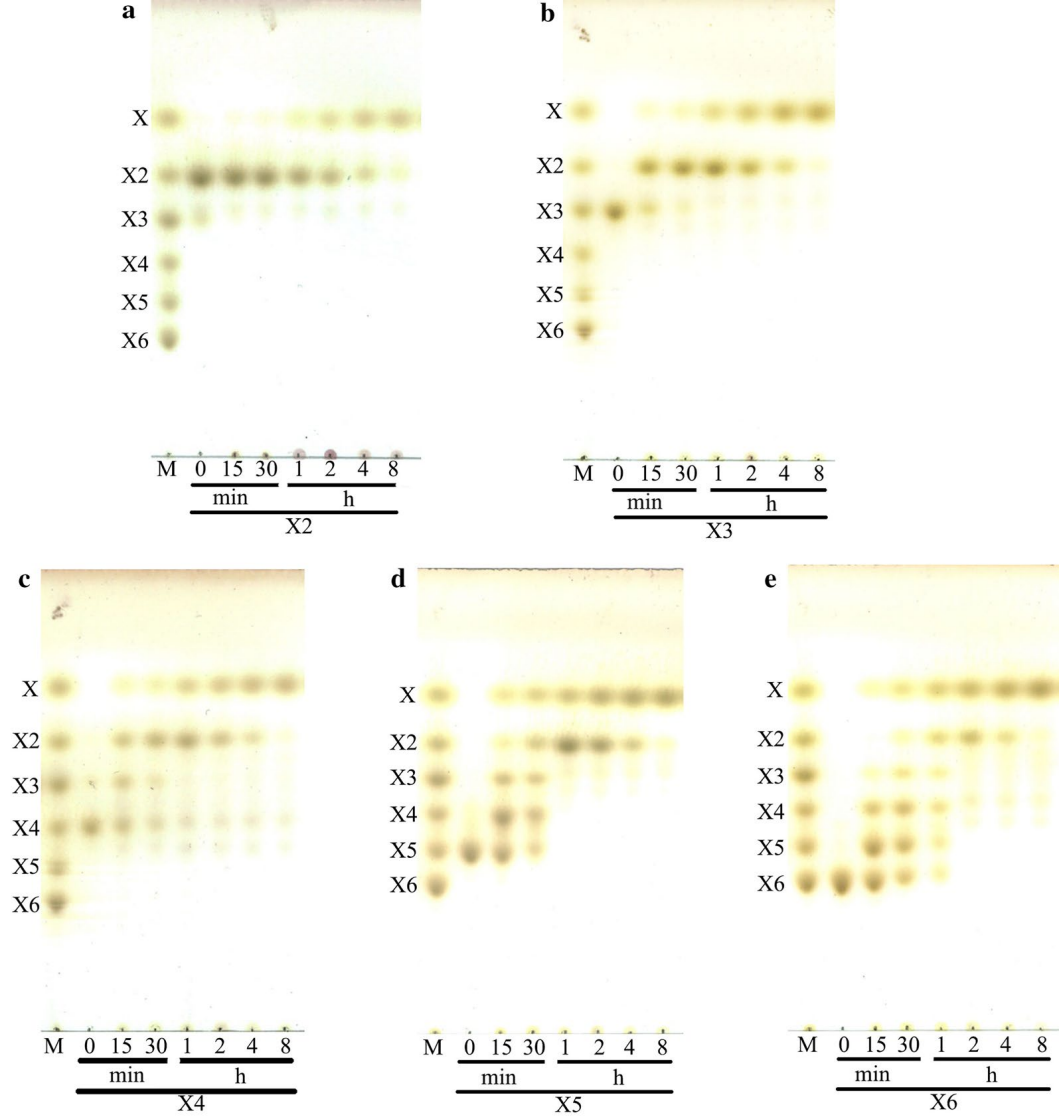
TLC analysis of hydrolysis products of various substrates by PbRex8. **a** Xylose; **b** xylobiose; **c** xylotriose; **d** xylotetraose; **e** xylopentaose; **f** xylohexaose. *M* marker, *X* xylose, *X2* xylobiose, *X3* xylotriose, *X4* xylotetraose, *X5* xylopentaose, *X6* xylohexaose

PbRex8 hydrolyzed XOSs (DP 3–6) to release mainly xylose and xylobiose at the initial 1 h, and the formed xylobiose was then further converted to xylose with the extension of incubation time (Fig. 4). PbRex8 could not hydrolyze *p*NP-β-xylopyranoside, reduced xylotriose and reduced xylotetraose (Additional file 1: Fig. S2a and b), suggesting that it catalyzed the release of xylose from the reducing end of XOSs. PbRex8 hydrolyzed birchwood xylan and oat β-glucan to release XOSs with DP 4–6 and glucooligosaccharides (GOSs) with DP 2–5, respectively (Additional file 1: Fig. S2c, d).

### Kinetic and inhibition constants of PbRex8

The *K*_m_ and *V*_max_ values of PbRex8 towards X2, X3 and X4 were determined to be 69.8 ± 4.4 mM and 137.6 ± 4.1 μmol/min/mg, 13.1 ± 0.8 mM and 522.4 ± 15.6 μmol/min/mg, and 9.8 ± 0.5 mM and 550.1 ± 12.8 μmol/min/mg, respectively. In addition, PbRex8 was competitively inhibited by xylose with a *K*_i_ value of 12.9 ± 0.6 mM.

### Production of xylose from corncobs by PbXyn10A co-action with PbRex8 or a characterized β-xylosidase

**Fig. 5:**
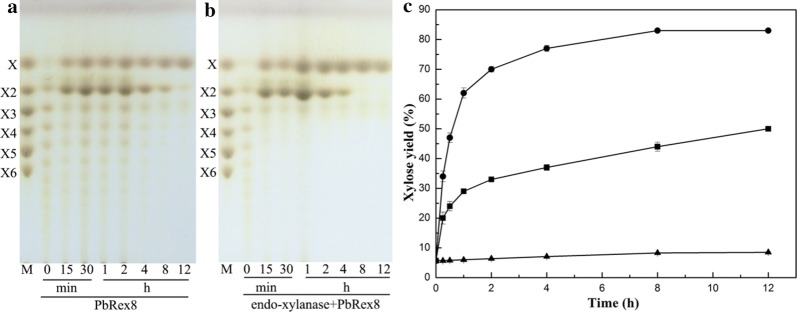
Time course profile for xylose production from corncobs by the co-action of PbRex8 and PbXyn10A. TLC analysis of the hydrolysis products of SEMC by PbRex8 alone (**a**) or PbRex8 coupled with PbXyn10A  (**b**); lanes M, standards containing xylose (X), xylobiose (X2), xylotriose (X3), xylotetraose (X4), xylopentaose (X5) and xylohexaose (X6). Incubation time (h or min) and substrates are indicated. **c** HPLC analysis of xylose production from SEMC by PbRex8 and PbXyn10A either alone or combination. Symbols: PbXyn10A (filled triangle); PbRex8 (filled square); PbRex8 coupled with PbXyn10A (filled circle). The values are the average of experiments performed in triplicate

The xylose yields of 50% ± 0.6 (w/w), 8.5% ± 0.2 (w/w) and 83% ± 0.3 (w/w) were obtained from steam explosion mixtures of corncobs (SEMC, containing of 5.7% ± 0.9 xylose (w/w)) by PbRex8, a xylanase from *P. barengoltzii* (PbXyn10A), and combination of PbRex8 and PbXyn10A for 12 h of incubation, separately (Fig. 5). The xylose yields of 33% ± 1.1 (w/w) and 80% ± 0.5 (w/w) were obtained from SEMC hydrolyzed by a β-xylosidase (PtXyl43) from *Paecilomyces thermophila* and PtXyl43 co-action with PbXyn10A (Additional file 1: Table S1). The hydrolysis products of SEMC by PtXyl43, PbRex8, PtXyl43 co-action with PbXyn10A and combination of PbRex8 and PbXyn10A were mainly xylose with contents (w/w) of 75%, 91%, 94.8% and 97.6%, respectively, while mainly X2, X3 and X4 by PbXyn10A (Additional file 1: Table S1).

### Crystal structure of PbRex8

The crystal structure of PbRex8 was determined at 1.88 Å resolution in space group *P*1 with four molecules in the asymmetric unit. The *R*_work_ and *R*_free_ were 24.42% and 28.91%, respectively (Additional file 1: Table S2). The structure of PbRex8 comprised a disordered (*α/α*)_6_ barrel formed by six inner and six outer α helices. A putative catalytic acid (Glu69) and a catalytic base (Asp261) were found in the middle of the catalytic cleft (Additional file 1: Fig. S3).

**Fig. 6:**
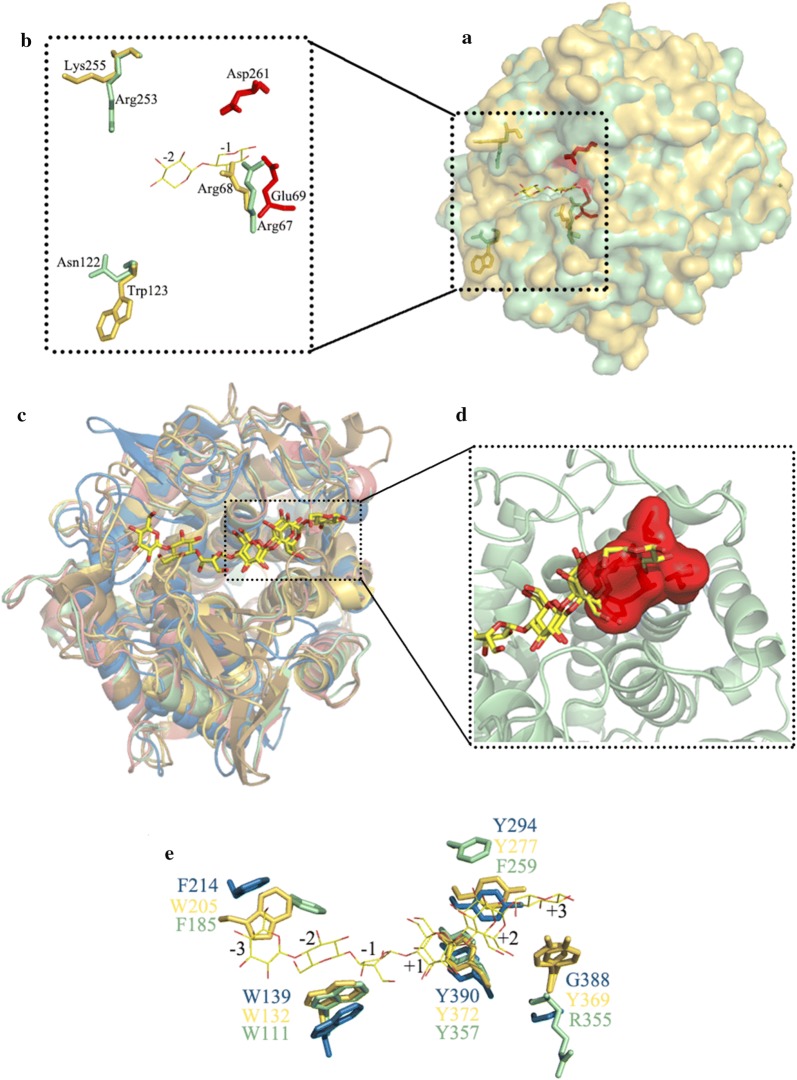
Structural comparison of PbRex8 with other GH family 8 enzymes. **a** PbRex8 in pale green (PDB code: 5YXT) and BhaRex8 in yellow orange (PDB code: 1WU4). **b** Residues from PbRex8 in pale green (Arg67, Asn122 and Arg253); residues from BhaRex8 in yellow orange (Arg68, Trp123 and Lys255). Two catalytic residues (Glu69 and Asp261) from PbRex8 are shown by red sticks. **c** PbRex8 in pale green (PDB code: 5YXT), BhaRex8 in salmon (PDB code: 1WU4), PhXyl8 in sand (PDB code: 1H12), Cel8A in yellow orange (PDB code: 1KWF), BGlc8H in sky blue (PDB code: 5Xd0). **d** The difference of Ala315-Pro318 in PbRex8 leads to the space steric hindrance. **e** protein–sugar stacking interactions along the substrate-binding cleft (PbRex8, BGlC8H and Cel8A) are shown in stick diagram: PbRex8 in pale green, Cel8A in yellow orange and PhXyl8 in sand

Superimposition of the structures revealed that PbRex8 shared the highest overall fold similarity with 1WU4 from *B. halodurans* BhaRex8 (RMSD = 0.7) [23], followed by 1H12 from *Pseudoalteromonas haloplanktis* PhXyl8 (RMSD = 1.9) [24], 5xd0 from *Paenibacillus* sp. X4 PGlC8H (RMSD = 2.2) [25] and 1KWF from *Clostridium thermocellum* Cel8A (RMSD = 2.3) [26]. Through superimposition structures of PbRex8 and BhaRex8 (1WU6, a crystal structure complexed with xylobiose), putative key residues along with catalytic groove were found. His317 could directly hydrogen-bond with the β-hydroxyl of the xylose at subsite +1 in the structure of PbRex8, contributing to the discrimination of the anomers at the reducing end (Additional file 1: Fig. S3). Arg67 showed a close conformation to subsite -1 (Fig. 6a, b). Asn122 and Arg253 showed a close extension to subsite -3 (Fig. 6a, b). A loop (Ala315-Pro318) may form a blockage at subsite +2 (Fig. 6c, d). In addition, Trp205, Trp132, Tyr372, Tyr277 and Tyr369 in the grove of PbRex8 were supposed to involve in glucooligosaccharides interactions by superimposed PbRex8 and Cel8A (1KWF, a structure complex with substrate) (Fig. 6e).

### Site-directed mutagenesis and the enzyme properties of mutants

Three mutants (R67A, N122A and R253A) were successfully expressed in *E. coli* BL21 (DE3). The mutants were purified and verified by SDS-PAGE (Additional file 1: Fig. S4). The optimal pH and temperature of the three mutants were determined to be 5.5 and 55 °C, respectively (data not shown). R67A displayed maximal activity towards X4 (292 ± 6 U/mg), followed by X3 (276 ± 4 U/mg), and trace activity towards X2 (2.5 ± 0.3 U/mg). N122A and R253A showed the highest activity towards X3 (368 ± 8 U/mg, 356 ± 5 U/mg), followed by X4 (190 ± 4 U/mg, 178 ± 3 U/mg), X2 (70 ± 0.9 U/mg, 72 ± 1.1 U/mg) (Table 2).

**Table 2:** Substrate specificities of mutants

Substrate	Specific activity (U/mg)^a^		
	R67A	N122A	R253A
X2	2.5 ± 0.3	70 ± 0.9	72 ± 1.1
X3	276 ± 4	368 ± 8	356 ± 5
X4	292 ± 6	190 ± 4	178 ± 3

^a^The specific activity was determined by measuring the enzyme’s activity in 5 mM citrate buffer (pH 5.5) at 55 °C using X2, X3 and X4 as the substrates

## Discussion

Xylanases have drawn great attention in recent years due to their wide range of biotechnological applications, such as biofuel, food, pulp and paper, animal feed and textile fields [7, 12]. So far, a number of microbial endo-xylanases have been identified and characterized [27], but there is still less information on exo-xylanases [19, 28]. Moreover, no exo-xylanase has been ever reported from *P. barengoltzii.* In this study, a GH family 8 reducing-end xylose-releasing exo-oligoxylanase (PbRex8) from *P. barengoltzii* CAU904 was identified and biochemically characterized.

Currently, only four GH family 8 reducing-end xylose-releasing exo-oligoxylanases (Rexs) have been reported [16–19]. Among these, only the sequence of Rex from *B. intestinalis* had a putative signal peptide [18]. No signal peptide was predicted in PbRex8, which is consistent with other three Rexs [16, 17, 19]. PbRex8 shared the highest sequence identity of 74% with the Rex from *B. halodurans* (Fig. 1), indicating that PbRex8 should be a new member of GH family 8 Rexs. PbRex8 had a specific activity of 331 ± 3 U/mg, which is obviously higher than that of Rexs from *B. halodurans* (84 U/mg) [16] and *P. barcinonensis* (146 U/mg) [19].

PbRex8 was an acidic Rex with an optimal pH of 5.5 (Fig. 3a), which is lower than those of other four Rexs from *B. halodurans* (6.2–7.3) [16], *B. intestinalis* (6.0) [18], *B. adolescentis* (6.0) [17] and *P. barcinonensis* (7.0) [19]. Acidic property is typically preferred in biorefinery due to that biomasses are usually pretreated in acidic conditions prior to enzymatic hydrolysis. PbRex8 exhibited an optimal temperature of 55 °C (Fig. 3c), which is higher than that of the other four Rexs having optimal temperatures in the range of 30–50 °C [16–19]. The half-life of PbRex8 at 50 °C was 380 ± 15 min, which is advantageous for bioconversion processes (Fig. 3e).

PbRex8 is distinct from the four previously reported Rexs in substrate specificity. It exhibited high activity towards xylobiose, while the others did not [16–19]. In addition, PbRex8 was most active on xylotetraose, but the other four Rexs were most active on xylotriose [16–19]. However, the *K*_m_ value of PbRex8 toward XOS (e.g., 13.1 ± 0.8 mM toward X3) was higher than those of Rexs from *B. halodurans* (2.4 ± 0.2 mM toward X3) [16] and *P. barcinonensis* (1.64 ± 0.03 mM toward X3) [19]. Therefore, branched XOSs may be the optimal substrates for PbRex8. Comparison of the structure of PbRex8 with BhRex8 indicated that Arg67 in the catalytic groove of PbRex8 is closer to subsite -1 than that of BhRex8 (Fig. 6b). A mutant R67A was designed, and this mutant almost abolished xylobiase activity (Table 2). These results demonstrated that R67 is a key residue for PbRex8 having xylobiase activity. The non-reducing end in catalytic groove of PbRex8 presented a more open state when compared to that of BhaRex8, this may be one of the reasons that PbRex8 exhibited higher activity on XOSs than that of BhRex8 (Fig. 6a). PbRex8 showed the highest specific activity on xylotetraose rather than xylotriose (Table 1), which may be due to that Asn122 and Arg253 in the catalytic groove of PbRex8 extended to subsite -3. Both of the two mutants (N122A and R253A) displayed maximal activity towards X3, which is different from that of PbRex8. These results demonstrated that N122 and R253 could be involved in hydrogen bonding with subsite -3 (Fig. 6b). PbRex8 was inactive on *p*NP-X, reduced xylobiose and reduced xylotetraose, which is accordance to that of the other four reported Rexs [16–19].

PbRex exhibited low activity on various xylans, which is similar to that of the Rexs from *P. barcinonensis* [19] and *B. adolescentis* [17], but different from that (showed no activity on xylans) of the Rexs from *B. halodurans* [16] and *B. intestinalis* [18]. Interestingly, PbRex8 displayed activity on β-1,3-1,4 glucan (1.3 U/mg towards oat β-glucan) (Table 1), the property of which has not been reported for Rexs yet. Herein, we superposed the structures of PbRex8, PhXyl8, Cel8A and GlC8H [24–26], the results revealed that the four overall structures shared a similar catalytic groove (Fig. 6c). Trp205, Trp132, Tyr372, Tyr277 and Tyr369 of PbRex8 were supposed to bound sugars at subsites − 3, − 2, + 1, + 2 and + 3, respectively, by stacking interaction residues of PbRex8 and Bglc8 (Fig. 6e). The recognition mechanism of PbRex8 is similar to that of Cel8A [26, 29]. PbRex8 and other enzymes displayed significant difference in the substrate-binding cleft, viz. Ala315-Pro318 in the structure of PbRex8 formed a blockage of subsite + 2 in catalytic cleft, while the others did not (Fig. 6d). The blockage sequence may contribute to the low activities of PbRex8 on xylans and β-1,3-1,4 glucans.

The action pattern of PbRex8 is consistent with that of the characterized Rexs, where the oligosaccharides are progressively degraded from *X*_n_ to *X*_n−1_, and the intermediate products are then further degraded in the same manner [16–19]. However, the final products were quite different, as PbRex8 hydrolyzed XOSs to release xylose as sole end product, while the other four Rexs hydrolyzed XOSs to yield mainly xylose and xylobiose. In addition, it is worth noting that PbRex8 could not hydrolyze reduced xylotriose and reduced xylotetraose (β-xylosidases did), indicating the hydrolysis properties of PbRex8 is different from that of β-xylosidases [2, 9–11].

Corncobs, as one of the most abundant renewable agriculture wastes (nearly 46 million tons annually) in china, contain approximately 35% (w/w) of xylan, showing great potential for biofuel and xylitol production [5, 21]. In the bioconversion process, xylan should be degraded to xylose first, and the products were subsequently converted to ethanol by fermentation or other chemicals [3, 6]. Traditionally, xylose was produced from corncobs by strong acid hydrolysis, which may bring environmental pollution risk [30–32]. Hence, development of green bioprocesses for xylose production is of great value. As PbRex8 coupled with a xylanase could catalyze the release of xylose efficiently from XOSs, it was applied in xylose production from corncobs by the synergistic reaction, and the highest conversion ratio of 83% ± 0.3 (w/w) was achieved. Usually, the complete conversion of biomass xylan to xylose requires the synergistic action of endo-xylanase and exo-β-xylosidase [33, 34]. Thus, a characterized β-xylosidase (PtXyl43) co-action with PbXyn10A was also used to evaluate xylose production from SEMC. The highest xylose yield of 80% ± 0.5 (w/w) was obtained, which is a little lower than that by the combination of PbRex8 and PbXyn10A. In addition, several attempts have been carried out for xylose production from different biomasses by endo-xylanases and exo-β-xylosidases, but most of the xylose yields are lower than that in the present study (Table 3). Therefore, PbRex8 could be a good candidate for xylose green production in biofuel industry.

**Table 3:** Summary of representative xylose productions from different lignocellulosic materials

Xylan source	Conversion method	Xylose yield (%)^a^	References
Chemical method			
Corn stover	Dilute acid hydrolysis	73.5 ± 1.5	[30]
Miscanthus	Weak-acid surface sites	74.1^b^	[31]
Napier grass	Hydrothermal process with phosphoric acid	77.2 ± 2.2	[32]
Corncobs	Dilute acid hydrolysis	85.4^b^	[5]
Enzymatic method			
Corncobs	Endo-xylanase and Rex (*P. barengoltzii*), steam explosion using AEW	83 ± 0.3	This study
Corncobs	Endo-xylanase (*P. barengoltzii*) and β-xylosidase (*P. thermophila*), steam explosion using AEW	80 ± 0.5	This study
*Eucalyptus grandis* wood xylan	Endo-xylanase and β-xylosidase (*A. pullulans*), alkali extraction	81.2 ± 1.5	[35]
Beechwood xylan	Endo-xylanase (*A. flavithermus*) and β-xylosidase (*S. solfataricus*)	63.6^b^	[46]
Corncobs	Endo-xylanase and β-xylosidase (*C. clariflavum*), alkali extraction	60.9^b^	[36]
Oat spelt xylan	Endo-xylanase and β-xylosidase (*P. janczewskii*)	42.8^b^	[11]
Barely straw	Endo-xylanase and β-xylosidase (*P. chrysosporium*), alkali extraction	7.4 ± 0.03	[10]
Wheat bran	Endo-xylanase and β-xylosidase (*T. thermophiles*), alkali extraction	6.3^b^	[9]

^a^The xylose yield was calculated based on xylan content in biomass

^b^Errors or deviations were not mentioned

## Conclusions

A novel reducing-end xylose-releasing exo-oligoxylanase (PbRex8) from *P. barengoltzii* was identified and characterized. PbRex8 was most active at pH 5.5 and 55 °C, respectively. The enzyme exhibited a unique hydrolysis property of degrading XOSs to xylose from the reducing end. PbRex8 efficiently converted corncobs to xylose with a high yield of 83% by combination with an endo-xylanase, exhibiting great application potential in biorefinery industries. Moreover, the crystal structure of PbRex8 was resolved and the mechanism underlying the unique substrate specificity was elucidated, which may be helpful for further molecular modification in terms of industrial use.

## Materials and methods

### Strain and reagents

*Paenibacillus barengoltzii* CAU904 used in this study has been preserved in the China General Microbiological Culture Centre (CGMCC) under accession No. 9530. *E. coli* strains DH5α and BL21 (DE3) were used as hosts for gene cloning and expression, respectively. pET-28a(+) was obtained from Novagen (Madison, WI, USA). LA Taq DNA polymerase was the product of Takara (Dalian, China). T4 DNA ligase and the restriction endonucleases were obtained from New England Biolabs (Ipswich, MA, USA).

Birchwood xylan, beechwood xylan, oat spelt xylan, barely β-glucan, oat β-glucan, lichenin, carboxymethylcellulose (CMC, low viscosity), locust bean gum and *p*NP-β-xylopyranoside were purchased from Sigma (St Louis, USA). Xylose (X), X2–X6 were purchased from Megazyme (Bray, Ireland). Reduced xylotriose and xylotetraose were prepared as the method described in the previous study [37]. Ni-IDA (Chelating Sepharose Fast Flow) and Sephacryl S-100 HR resins were from GE Life Sciences (NJ, USA). Thin layer chromatographic (TLC) silica gel plates were obtained from E. Merck (Darmstadt, Germany).

### Cloning and sequence analysis of a Rex gene

Recombinant DNA techniques were performed as described by Sambrook and Russell [38]. *P. barengoltzii* CAU904 was cultured as described by Yang et al. [39]. Genomic DNA of *P. barengoltzii* CAU904 isolated by an Easy-pure Bacteria DNA kit was used as template for polymerase chain reaction (PCR) amplification. To clone a Rex gene, specific primers PbRex8F (5′ATGAAGGAGCATCAAGGG3′) and PbRex8R (5′TTACGCCTCACCCCTC3′) were designed. PCR conditions were as follows: step 1—hot start at 94 °C for 5 min, step 2—35 cycles of 94 °C for 30 s, step 3—54 °C for 30 s, step 4—72 °C for 3 min, and step 5—5 min of extension at 72 °C. The PCR product was purified, ligated to pMD19-T vector and verified by sequencing.

### Expression of PbRex8 in *E. coli*

The coding region of the Rex gene (*PbRex8*) was amplified by PCR using the primers *Pb*RexNheF (5′CTCAGGCTAGCATGAAGGAGCATCAAGGG3′, *Nhe* I restriction site is underlined) and *Pb*Rex8XolR (5′CTCAGCTCGAGTTACGCCTCACCCCTC3′, *Xol*I restriction site is underlined). The PCR product was purified by gel kit (Biomed, Beijing), digested by restriction enzymes *Nhe* I and *Xol* I, and then subcloned into the corresponding sites of the pET28a(+) vector which was digested by the same enzymes. The recombinant vector with a His_6_-tag at its N terminal was transformed into *E. coli* BL21 competent cells for protein expression.

The positive colonies were screened on LB agar-plates containing kanamycin (50 μg/mL). A single colony of *E. coli* BL21 harboring the recombinant plasmid of pET28a(+)-*Pb*Rex8 was inoculated into LB medium containing 50 μg/mL of kanamycin and incubated in a rotary shaker (200 rpm) at 37 °C. When the absorbance of the broth at 600 nm reached 0.6–0.8, IPTG was added to the broth to a final concentration of 1 mM for induction, and the culture was further grown at 20 °C for 12 h.

### Purification of PbRex8

The cells were collected by centrifugation (12,000×*g*, 10 min), suspended in buffer A (20 mM Tris–HCl pH 8.0, 20 mM imidazole, 500 mM NaCl), and then disrupted by sonication. The suspension was centrifuged at 12,000×*g* for 10 min, and the supernatant was harvested and loaded onto a Ni-IDA column pre-equilibrated with buffer A at a flow rate of 0.5 mL/min. After binding for 30 min, the unbound and weakly bound impurities were washed with buffer A and buffer B (20 mM Tris–HCl pH 8.0, 50 mM imidazole, 500 mM NaCl), separately. The bound proteins were eluted with buffer C (20 mM Tris–HCl pH 8.0, 200 mM imidazole, 500 mM NaCl) at a flow rate of 1.0 mL/min. The eluted fractions having Rex activities were combined and checked for homogeneity by sodium dodecyl sulfate-polyacrylamide gel electrophoresis (SDS-PAGE).

### Enzyme assay and protein concentration determination

The Rex activity was determined by calculating the xylose-releasing rate from X3 [19]. The reaction mixture containing 90 μL of 1.0% (w/v) xylotriose and 10 μL of suitably diluted enzyme solution (5–15 μg/mL) in 5 mM citrate buffer (pH 5.5) was incubated at 55 °C for 10 min. The amount of xylose released was quantified by high-performance liquid chromatography (HPLC) equipped with a refractive index detector (RID) and a sugar column (Shodex Sugar KS-802). The column was maintained at 65 °C and eluted with deionized water at a flow rate of 0.8 mL/min. One unit (U) of Rex activity was defined as the amount of enzyme liberating 1 μmol of xylose per minute under the above assay conditions. The protein concentration was determined by Lowry method using bovine serum albumin as the standard [40].

### SDS-PAGE and molecular mass determination

SDS-PAGE was carried out as described by Laemmli [41] using 12.5% separating gel and 4.0% stacking gel. The protein bands were stained with Coomassie brilliant blue R-250. The native molecular weight of PbRex8 was determined by gel filtration using Sephacryl-100 column (1 cm × 40 cm) equilibrated with 20 mM citrate (pH 5.5) containing 150 mM NaCl at a flow rate of 0.33 mL/min. The protein standards used were cytochrome c (12.4 kDa), α-chymotrypsinogen a (from bovine pancreas, 25.6 kDa), ovalbumin (44.3 kDa), bovine serum albumin (66.5 kDa) and phosphorylase b (97.2 kDa).

### Biochemical properties of recombinant PbRex8

The optimal pH of PbRex8 was determined in various buffers (5 mM) within pH 3.0–11.0. The buffers used included citrate buffer (pH 3.0–6.0), acetate buffer (pH 4.0–6.0), 2-(morpholino)ethanesulfonic acid (MES) buffer (pH 5.5–6.5), 2-morpholinopropanesulfonic acid (MOPS) buffer (pH 6.5–7.5), Tris–HCl buffer (pH 7.0–9.0), 2-cyclohexylaminoethanesulfonic acid (CHES) buffer (pH 8.0–10.0), glycine–NaOH buffer (pH 9.0–10.5) and *N*-cyclohexyl-3-aminopropanesulfonic acid (CAPS) buffer (pH 10.0–11.0). To determine the pH stability, the enzyme samples were incubated in the above-mentioned buffers at 50 °C for 30 min, and the residual activities were then determined by the standard assay.

The optimal temperature was determined at different temperatures (20–80 °C) in 5 mM citrate buffer (pH 5.5). Thermostability was determined by evaluating the residual enzyme activity after 30 min pre-incubation in 5 mM citrate buffer (pH 5.5) at different temperatures (20–80 °C). The thermal inactivation of PbRex8 was estimated by incubating the enzyme in 5 mM citrate buffer pH 5.5 at 50, 55 and 60 °C for 8 h, respectively.

The influence of metal ions and other agents on the activity of PbRex8 were evaluated. The enzyme was incubated in 5 mM citrate buffer pH 5.5 in the presence of 1 mM of metal ions including K^+^, Na^+^, Ag^+^, Fe^2+^, Co^2+^, Ca^2+^, Cu^2+^, Zn^2+^, Mn^2+^, Hg^2+^, Ni^2+^, Mg^2+^, Ba^2+^ and Fe^3+^, as well as other reagents including 1 mM EDTA, SDS and β-mercaptoethanol at 50 °C for 30 min, followed by incubation at 0 °C for 30 min. The residual activities were then determined by the standard assay.

### Substrate specificity and hydrolysis properties of PbRex8

Substrate specificity of PbRex8 was determined in 20 mM citrate buffer (pH 5.5) at 55 °C for 10 min using various substrates. The tested polysaccharides (1% w/v) included birchwood xylan, beechwood xylan, oat spelt xylan, β-glucan (from barely), oat β-glucan, lichenin, locust bean gum and carboxymethylcellulose (CMC, low viscosity). The reaction mixture contained 900 μL of 1% (w/v) different substrates in 20 mM citrate buffer (pH 5.5) and 100 μL of appropriately diluted enzyme solution (0.2–0.8 mg/mL). The amount of released reducing sugars was measured by DNS method [42]. For *p*NP-β-xylopyranoside, 50 μL of suitably diluted enzyme solution (1 mg/mL) was added into 250 μL of substrate solution (5 mM) in 20 mM citrate buffer (pH 5.5), and incubated at 55 °C for 10 min. The amount of formed *p*NP was determined by spectrophotometry at 410 nm. For XOSs with DP 2–6, the enzyme activities were determined according to the standard enzyme assay, and the enzyme concentrations for X2, X4, X5 and X6 were 100, 10, 15 and 20 μg/mL, respectively. One unit of enzyme activity was defined as the amount of enzyme required to produce 1 μmol reducing sugar, or *p*NP, or xylose per minute under the above assay conditions.

The hydrolysis properties of PbRex8 towards XOSs (DP 2–6), reduced xylotriose (X3r), reduced xylotetraose (X4r), birchwood xylan and oat β-glucan were investigated by analyzing the hydrolysis products using TLC method. A total of 1 mL of reaction mixture containing 1% (w/v) various substrates and 50 U PbRex8 was incubated at 50 °C for 8 h, separately. For birchwood xylan and oat β-glucan, 5 U/mL of enzyme was added. Aliquots withdrawn at different time intervals were immediately boiled for 10 min, and then subjected to TLC analysis. The samples were spotted onto a TLC silica gel plate (Merck, Darmstadt, Germany), developed twice in a butanol-acetic acid–water (2:1:1, v/v/v) solvent system. Saccharides were detected by immersing the plates in solution containing methanol:sulfuric acid (95:5, v/v) for few seconds, followed by heating in an oven. Mixtures of X1–X6 were used as the standards.

### Kinetic and inhibition constants of PbRex8

The initial rates of PbRex8 towards X2 (35–110 mM), X3 (7–22 mM) and X4 (5–17.5 mM) were analyzed by determining the enzyme activities in 5 mM citrate buffer pH 5.5 at 55 °C for different times (1–10 min), and the results suggested that reactions progress linearly in 5 mM citrate buffer pH 5.5 at initial 5 min (Fig. 6). Thus, the kinetic parameters of PbRex8 were determined by measuring the enzyme activity in 5 mM citrate buffer pH 5.5 at 55 °C for 5 min with different substrate concentrations (5–110 mM). The substrate concentrations of X2, X3 and X4 were in the ranges of 35–110, 7–22 and 5–17.5 mM, respectively, and the correspondent enzyme concentrations were in the ranges of 50–200, 5-15 and 5–15 μg/mL, respectively. To determine *K*_i_ value of PbRex8 for xylose, the enzyme activity of PbRex8 was measured using X3 as the substrate in the presence of 5–20 mM xylose in 5 mM citrate buffer pH 5.5 at 55 °C for 5 min according to the standard enzyme assay. The kinetic parameters and inhibition constants were calculated by nonlinear regression fit of Michaelis–Menten with GraphPad Prism software [22].

### Xylose production from corncobs

The corncobs for xylose production contained 31.2% (w/w) hemicellulose, 45.6% (w/w) cellulose and 9.9% (w/w) lignin (Additional file 1: Table S3). Before xylose production, corncobs were pretreated by steam explosion using acidic electrolyzed water (pH 2.0) and the endogenous xylanase (PbXyn10A) from *P. barengoltzii* were prepared according to previous study [21]. A characterized β-xylosidase (PtXyl43) from *Paecilomyces thermophila* was prepared according to the previous literature [33, 43]. A reaction mixture containing 25 mL steam explosion mixture of corncobs (SEMC pH 5.5) was incubated at 50 °C in the presence of PbRex8 (50 U/mL), PtXyl43 (5 U/mL) and PbXyn10A (50 U/mL) either alone or in combination for 12 h, samples were periodically taken and boiled for 10 min to terminate the reaction. Before analysis, the water-insoluble fraction was removed by filtration and the filter residue was washed 3 times by distilled water. The filter liquor was collected, qualitatively and quantitatively analyzed by TLC and HPLC, respectively. The xylose yield is the percentage of released xylose weight (g) to initial xylan in raw corncob weight (g). The xylose content is the percentage of released xylose weight (g) in the hydrolysate to the released total sugars (g) in the hydrolysate.

### Crystallization, data collection and structure determination

Crystallization was carried out using the sitting-drop vapor diffusion method. PbRex8 solution was concentrated to 10 mg/mL in 20 mM citrate buffer pH 6.0, and then screened at 20 °C by crystallization solution kits (Hampton Research, USA). Crystals suitable for diffraction were grown in 0.2 M sodium thiocyanate and 20% polyethylene glycol (PEG) 3350. Crystals were soaked in reservoir solution supplemented with 20% glycerol and then vitrified in liquid nitrogen. Diffraction data of PbRex8 were collected at beamline BL17U at Shanghai Synchrotron Research Facility (SSRF, China). The collected diffraction data were processed with HKL-2000 [44].

The structure of PbRex8 (PDB ID: 5YXT) was resolved by molecular replacement using *B. halodurans* reducing-end xylose-releasing exo-oligoxylanase (PDB ID: 1WU4) as a search model. The structure model was built and refined with the Phenix suite [45]. The detailed statistics of data collection and refinement are shown in Additional file 1: Table S1.

### Site-directed mutagenesis and enzymatic properties

Mutants R67A, N122A and R253A were performed in PbRex8 using the Fast Mutagenesis System site-directed mutagenesis kit (TransGen Biotech, China) with the primers listed in Additional file 1: Table S4. All transformants were confirmed by DNA sequencing. The mutated enzymes were prepared in the same way as the wild-type enzyme. The enzymes properties of R67A, N122A and R253A were also determined in the same way as PbRex8.

## Additional file

**Additional file 1.** Additional tables and figures.

## Data Availability

All data generated or analyzed during this study are included in this article and its additional file. Any other data related to this manuscript will be made available by the corresponding author upon reasonable request.

## References

[1] Victor DG, Leape JP (2015). Global climate agreement: after the talks. Nature.

[2] Biely P, Singh S, Puchart V (2016). Towards enzymatic breakdown of complex plant xylan structures: state of the art. Biotechnol Adv.

[3] Tuck CO, Perez E, Horvath IT, Sheldon RA, Poliakoff M (2012). Valorization of biomass: deriving more value from waste. Science.

[4] Scheller HV, Ulvskov P (2010). Hemicelluloses. Annu Rev Plant Biol.

[5] Jiang LQ, Wu NN, Zheng AQ, Zhao ZL, He F, Li HB (2016). The integration of dilute acid hydrolysis of xylan and fast pyrolysis of glucan to obtain fermentable sugars. Biotechnol Biofules..

[6] Lyczakowski JJ, Wicher KB, Terrett OM, Faria-Blanc N, Yu X, Brown D, Krogh KBRM, Dupree P, Busse-Wicher M (2017). Removal of glucuronic acid from xylan is a strategy to improve the conversion of plant biomass to sugars for bioenergy. Biotechnol Biofules..

[7] You S, Chen CC, Tu T, Wang XY, Ma R, Cai HY, Guo RT, Luo HY, Yao B (2018). Insight into the functional roles of Glu175 in the hyperthermostable xylanase XYL10C-Delta N through structural analysis and site-saturation mutagenesis. Biotechnol Biofules..

[8] Ye YX, Li XZ, Cao Y, Du J, Chen SC, Zhao J (2017). A β-xylosidase hyper-production *Penicillium oxalicum* mutant enhanced ethanol production from alkali-pretreated corn stover. Bioresour Technol.

[9] Guerfali M, Maalej-Achouri I, Belghith H (2013). Hydrolytic potential of *Talaromyces thermophilus* β-xylosidase and its use for continuous xylose production. Food Technol Biotechnol..

[10] Nguyen DH, Cu LN, Seo J, Kim D, Park S (2015). Putative endoglucanase PcGH5 from *Phanerochaete chrysosporium* is a β-xylosidase that cleaves xylans in synergistic action with endo-xylanase. J Biosci Bioeng.

[11] Fanchini Terrasan CR, Trobo-Maseda L, Moreno-Perez S, Cano Carmona E, Costa Pessela B, Manuel Guisan J (2016). Co-immobilization and stabilization of xylanase, β-xylosidase and α-l-arabinofuranosidase from *Penicillium janczewskii* for arabinoxylan hydrolysis. Process Biochem.

[12] Juturu V, Wu JC (2012). Microbial xylanases: engineering, production and industrial applications. Biotechnol Adv.

[13] Collins T, Gerday C, Feller G (2005). Xylanases, xylanase families and extremophilic xylanases. FEMS Microbiol Rev.

[14] Pollet A, Delcour JA, Courtin CM (2010). Structural determinants of the substrate specificities of xylanases from different glycoside hydrolase families. Crit Rev Biotechnol.

[15] Honda Y, Kitaoka M (2004). A family 8 glycoside hydrolase from *Bacillus halodurans* C-125 (BH2105) is a reducing end xylose-releasing exo-oligoxylanase. J Biol Chem.

[16] Lagaert S, Pollet A, Courtin CM, Volckaert G (2014). β-Xylosidases and α-L-arabinofuranosidases: accessory enzymes for arabinoxylan degradation. Biotechnol Adv.

[17] Lagaert S, Van Campenhout S, Pollet A, Bourgois TM, Delcour JA, Courtin CM, Volckaert G (2007). Recombinant expression and characterization of a reducing-end xylose-releasing exo-oligoxylanase from *Bifidobacterium adolescentis*. Appl Environ Microb..

[18] Hong P, Iakiviak M, Dodd D, Zhang M, Mackie RI, Cann I (2014). Two new xylanases with different substrate specificities from the human gut bacterium *bacteroides intestinalis* DSM 17393. Appl Environ Microb..

[19] Valenzuela SV, Lopez S, Biely P, Sanz-Aparicio J, Pastor FIJ (2016). The glycoside hydrolase family 8 reducing-end xylose-releasing exo-oligoxylanase Rex8A from *Paenibacillus barcinonensis* BP-23 is active on branched xylooligosaccharides. Appl Environ Microb..

[20] Malgas S, Pletschke BI (2019). The effect of an oligosaccharide reducing-end xylanase, BhRex8A, on the synergistic degradation of xylan backbones by an optimised xylanolytic enzyme cocktail. Enzyme Microb Technol.

[21] Liu XQ, Liu Y, Jiang ZQ, Liu HJ, Yang SQ, Yan QJ (2018). Biochemical characterization of a novel xylanase from *Paenibacillus barengoltzii* and its application in xylooligosaccharides production from corncobs. Food Chem.

[22] Fu X, Yan QJ, Yang SQ, Yang XB, Guo Y, Jiang ZQ (2014). An acidic, thermostable exochitinase with β-*N*-acetylglucosaminidase activity from *Paenibacillus barengoltzii* converting chitin to *N*-acetyl glucosamine. Biotechnol Biofuels.

[23] Fushinobu S, Hidaka M, Honda Y, Wakagi T, Shoun H, Kitaoka M (2004). Structural basis for the specificity of the reducing end xylose-releasing exo-oligoxylanase from *Bacillus halodurans* C-125. J Biol Chem.

[24] Collins T, Meuwis MA, Stals I, Claeyssens M, Feller G, Gerday C (2002). A novel family 8 xylanase, functional and physicochemical characterization. J Biol Chem.

[25] Na HB, Jung WK, Jeong YS, Kim HJ, Kim SK, Kim J, Yun HD, Lee J, Kim H (2015). Characterization of a GH family 8 β-1,3-1,4-glucanase with distinctive broad substrate specificity from *Paenibacillus* sp X4. Biotechnol Lett.

[26] Alzari PM, Souchon H, Dominguez R (1996). The crystal structure of endoglucanase CelA, a family 8 glycosyl hydrolase from *Clostridium thermocellum*. Structure..

[27] Basu M, Kumar V, Shukla P (2018). Recombinant approaches for microbial xylanases: recent advances and Perspectives. Curr Protein Pept Sci.

[28] Mello BL, Alessi AM, Riano-Pachon DM, DeAzevedo ER, Guimaraes FEG, Espirito Santo MC, McQueen-Mason S, Bruce NC, Polikarpov I (2017). Targeted metatranscriptomics of compost-derived consortia reveals a GH11 exerting an unusual exo-1,4-β-xylanase activity. Biotechnol Biofuels.

[29] Guerin D, Lascombe MB, Costabel M, Souchon H, Lamzin V, Beguin P, Alzari PM (2002). Atomic (0.94 Å) resolution structure of an inverting glycosidase in complex with substrate. J Mol Biol.

[30] Shekiro JI, Kuhn EM, Nagle NJ, Tucker MP, Elander RT, Schell DJ (2014). Characterization of pilot-scale dilute acid pretreatment performance using deacetylated corn stover. Biotechnol Biofuels.

[31] Chung P, Charmot A, Olatunji-Ojo OA, Durkin KA, Katz A (2014). Hydrolysis of miscanthus xylan to xylose using weak-acid surface sites. ACS Catal..

[32] Takata E, Tsutsumi K, Tsutsumi Y, Tabata K (2013). Production of monosaccharides from napier grass by hydrothermal process with phosphoric acid. Bioresour Technol.

[33] Yan QJ, Wang L, Jiang ZQ, Yang SQ, Zhu HF, Li LT (2008). A xylose-tolerant β-xylosidase from *Paecilomyces thermophila*: characterization and its co-action with the endogenous xylanase. Bioresour Technol.

[34] Shi H, Li X, Gu HX, Zhang Y, Huang YJ, Wang LL, Wang F (2013). Biochemical properties of a novel thermostable and highly xylose-tolerant β-xylosidase/α-arabinosidase from *Thermotoga thermarum*. Biotechnol Biofules..

[35] Bankeeree W, Akada R, Lotrakul P, Punnapayak H, Prasongsuk S (2018). Enzymatic hydrolysis of black liquor xylan by a novel xylose-tolerant, thermostable β-xylosidase from a tropical strain of *Aureobasidium pullulans* CBS 135684. Appl Biochem Biotechnol.

[36] Geng A, Wang HC, Wu J, Xie RR, Sun JZ (2017). Characterization of a β-xylosidase from *Clostridium clariflavum* and its application in xylan hydrolysis. Bioresouces..

[37] Zhou P, Liu Y, Yan QJ, Chen Z, Qin Z, Jiang ZQ (2014). Structural insights into the substrate specificity and transglycosylation activity of a fungal glycoside hydrolase family 5 β-mannosidase. Acta Crystallogr Sect D.

[38] Sambrook J, Fritsch EF, Maniatis T (2001). Molecular cloning a laboratory manual.

[39] Yang SQ, Fu X, Yan QJ, Guo Y, Liu Z, Jiang ZQ (2016). Cloning, expression, purification and application of a novel chitinase from a thermophilic marine bacterium *Paenibacillus barengoltzii*. Food Chem.

[40] Lowry OH, Rosebrough NJ, Farr AL, Randall RJ (1951). Protein measurement with the folin phenol reagent. J Biol Chem.

[41] Laemmli UK (1970). Cleavage of structure proteins during the assembly of the head of bacteriophage T4. Nature.

[42] Miller GL (1959). Use of dinitrosalicylic acid reagent for determination of reducing sugars. Anal Chem.

[43] Teng C, Jia HY, Yan QJ, Zhou P, Jiang ZQ (2011). High-level expression of extracellular secretion of a β-xylosidase gene from *Paecilomyces thermophila* in *Escherichia coli*. Bioresour Technol..

[44] Otwinowski Z, Minor W (1997). Processing of X-ray diffraction data collected in oscillation mode, macromolecular cryatallography. Methods Enzymol.

[45] Adams PD, Afonine PV, Bunkoczi G, Chen VB, Davis IW, Echols N, Headd JJ, Hung L, Kapral GJ, Grosse-Kunstleve RW, McCoy AJ, Moriarty NW, Oeffner R, Read RJ, Richardson DC, Richardson JS, Terwilliger TC, Zwart PH (2010). PHENIX: a comprehensive Python-based system for macromolecular structure solution. Acta Crystallogr Sect D.

[46] Kambourova M, Mandeva R, Fiume I, Maurelli L, Rossi M, Morana A (2007). Hydrolysis of xylan at high temperature by co-action of the xylanase from *Anoxybacillus flavithermus* BC and the β-xylosidase/α-arabinosidase from *Sulfolobus solfataricus* O_α_. J Appl Microbiol.

