# Supplementary material for: Biochemical characterization of a novel exo-oligoxylanase from Paenibacillus barengoltzii suitable for monosaccharification from corncobs
Source: Biotechnol Biofuels. 2019 Jul 29;12:190. doi: 10.1186/s13068-019-1532-6 (PMC6661730; doi:10.1186/s13068-019-1532-6)
Supplement: Supplementary file 1 — Additional file 1. Additional tables and figures. [file 13068_2019_1532_MOESM1_ESM.docx]

**Additional materials**

**Biochemical characterization of a novel glycoside hydrolase family 8 exo-oligoxylanase from *Paenibacillus barengoltzii* suitable for monosaccharification of corncob xylan**

Xueqiang Liu ^1^, Zhengqiang Jiang ^2^, Yu Liu ^2^, Xin You ^2^, Shaoqing Yang ^2,*^, Qiaojuan Yan ^1, *^

^1^ Beijing Advanced Innovation Center for Food Nutrition and Human Health, College of Engineering, China Agricultural University, Beijing 100083, China

^2^ College of Food Science ＆ Nutritional Engineering, China Agricultural University, Beijing 100083, China

^*^ Corresponding authors. Tel.: +86 10 62737636; fax: +86 10 82388508. E-mail: [yanqj@cau.edu.cn](mailto:yanqj@cau.edu.cn) (Q.J. Yan); [ysq@cau.edu.cn](mailto:ysq@cau.edu.cn) (S.Q. Yang).

**Additional Table S1**

**X-ray data collection and refinement statistics.**

| **PbRex8** | |
| --- | --- |
| ***Data-collection statistics*** |  |
| Radiation source | SSRF-BL17U |
| Wavelength (Å) | 0.9788 |
| Resolution (Å) | 50-1.88(1.94-1.88) |
| Space group | *P*1 |
| Unit cell parameters |  |
| *a*, *b*, *c* (Å) | 54.6, 79.0, 89.2 |
| α, β, γ (°) | 100.3, 95.9, 106.4 |
| Protein molecules in asymmetric unit | 4 |
| Unique reflections | 105932 (10493) |
| Completeness (%) | 93.0 (86.3) |
| *R*_merge_*^a^* (%) | 7.4 (35.8) |
| Wilson B-factor (Å^2^) | 16.79 |
| ***Refinement statistics*** |  |
| Resolution (Å) | 22.4-1.88 |
| *R*_work_*^b^* (%) | 24.42 (35.54) |
| *R*_free_*^b^* (%) | 28.91 (37.24) |
| RMSD |  |
| Bond lengths (Å) | 0.007 |
| Bond angles (°) | 0.83 |
| Average B-factors (Å^2^) | 23.13 |
| Solvent | 31.73 |
| Ramachandran |  |
| Most favored regions (%) | 97.4 |
| Additional allowed regions (%) | 2.6 |
| Disallowed regions (%) | 0.07 |
| Clashscore | 8.45 |
| PDB code | 5YXT |

*^a^* *R*_merge_=$\sum_{hkl} \sum_{i} \left| Ii\left( hkl \right)-\left\langle I\left( hkl \right) \right\rangle\right|/\sum_{hkl} \sum_{i} Ii(hkl)$, where *Ii*(*hkl*) is the *i*th observation of reflection *hkl* and $\left\langle I\left( hkl \right) \right\rangle$ is the weighted average intensity for all observations *i* of reflection *hkl*

*^b^* *R*_work/free_=$\sum_{hkl} ||\mathrm{Fo}|-k|\mathrm{Fc}||/\sum_{hkl} |\mathrm{Fo}|$; 95% and 5% of reflections were used for *R*_work_ and *R*_free_, respectively

**Additional Table S2**

**The sugar compositions of corncobs and pretreated corncobs.**

| Compositions | Corncobs (100 g) | Pretreated Corncobs (58.5 g) |
| --- | --- | --- |
| Cellulose | 45.6±0.6 g | 45±0.5 g |
| Hemicellulose | 31.2±0.4 g | 9.9±0.8 g |
| Lignin | 9.9±0.1 g | 1.9±0.1 g |
| Others | 13.3±0.3 g | 1.7±0.1 g |

^a^ Yield data are mean values ± standard deviations of triplicate measurements.

**Additional Table S3**

**Xylose production from SEMC by enzymes^a^.**

| Enzyme | Xylose yield (%) | XOS yield (%) | Total yield (%) |
| --- | --- | --- | --- |
| PbXyn10A | 8.5±0.2 | 75±0.8 | 83.5 |
| PbRex8  PtXyl43 | 50±0.6  33±0.6 | 5±0.4  11±0.9 | 55  44 |
| PbRex8 and PbXyn10A  PtXyl43 and PbXyn10A | 83±0.3  80±0.5 | 2±0.2  4.4±0.3 | 85  84.4 |

^a^ Yield data are mean values ± standard deviations of quintuplicate measurements.

**Additional Table S4**

**Primers used in the site-directed mutagensis**

| Primer name | Primer sequence (5′→3′) ^a^ |
| --- | --- |
| R67A-UP | CCTGGACGTAGCAACGGAGGGGATGTCCTACGGGATG |
| R67A-DN  N122A-UP | TGCTACGTCCAGGTTGCCGGTATCCAGCATATAGCC  GCGTCTATCCGCTGGGCCGGCGCCAGATGGCGAGG |
| N122A-DN  R253A-UP | AGCGGATAGACGCTTGCCGTCCGGTGCACAGGACCAG  CAAATAACGAAGCGGGGTACGGCCACTTCTTCAG |
| R253A-DN | CGCTTCGTTATTTGGCGTTCCATCATAATATGC |

**^a^** The mutant sites are shadowed in grey.

**Additional Table S5**

**Optimal temperature and pH of PbRex8 and its mutants with xylotriose as the substrate**

| Enzyme^a^ | Optimal pH | Optimal temperature (^o^C) |
| --- | --- | --- |
| PbRex8 | 5.5 | 55 |
| R67A  N122A | 5.5  5.5 | 55  55 |
| R253A | 5.5 | 55 |

^a^ All measurements were performed in triplicate.

**
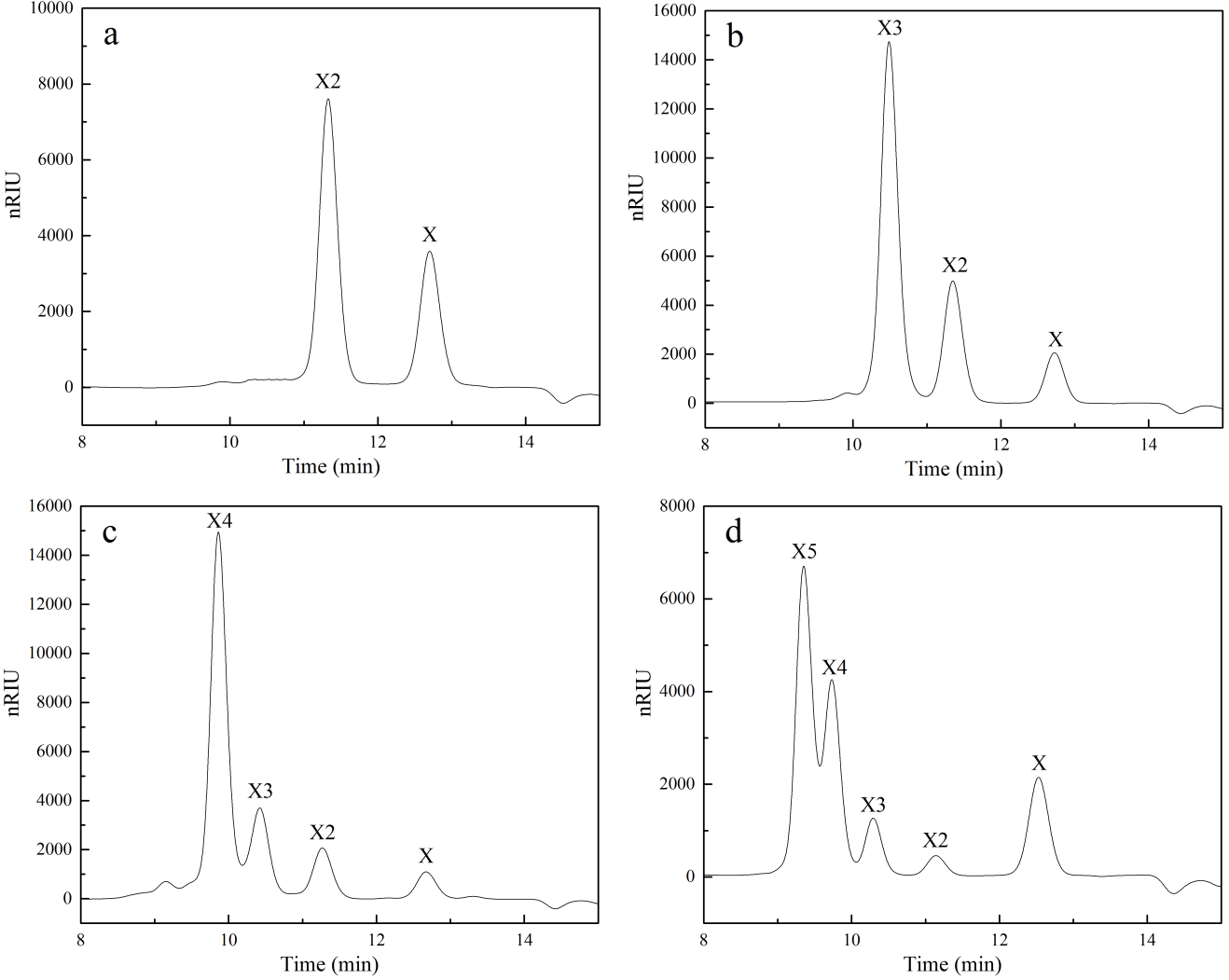
**

**Fig. S1.** HPLC analysis of enzyme activity determination for PbRex8 toward xylobiose (X2, a), xylotriose (X3, b), xylotetraose (X4, c) and xylopentaose (X5, d).


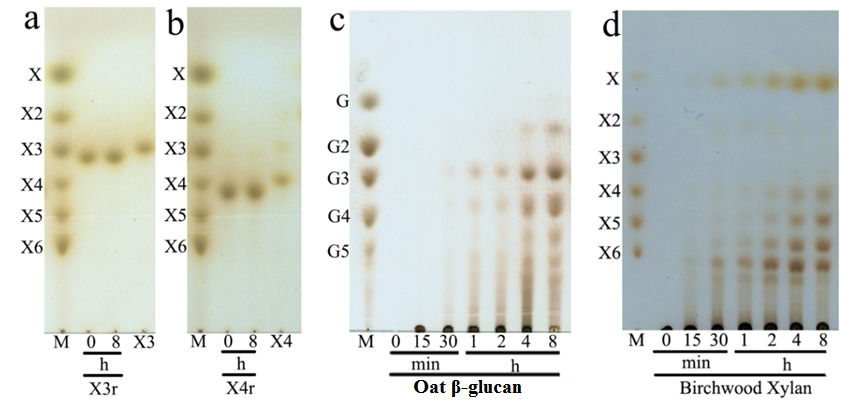


**Fig. S2.** TLC analysis of the hydrolysis products of X3r (a), X4r (b), oat β-glucan (c) and birchwood xylan (d) by PbRex8. Incubation times (h or min) are indicated. M: marker; X: xylose; X2: xylobiose; X3: xylotriose; X3r: reduced xylotriose; X4: xylotetraose; X4r: reduced xylotetraose; X5: xylopentaose; X6: xylohexaose; G: glucose; G2: cellobiose; G3: cellotriose; G4: cellotetraose; G5: cellopentose.

**
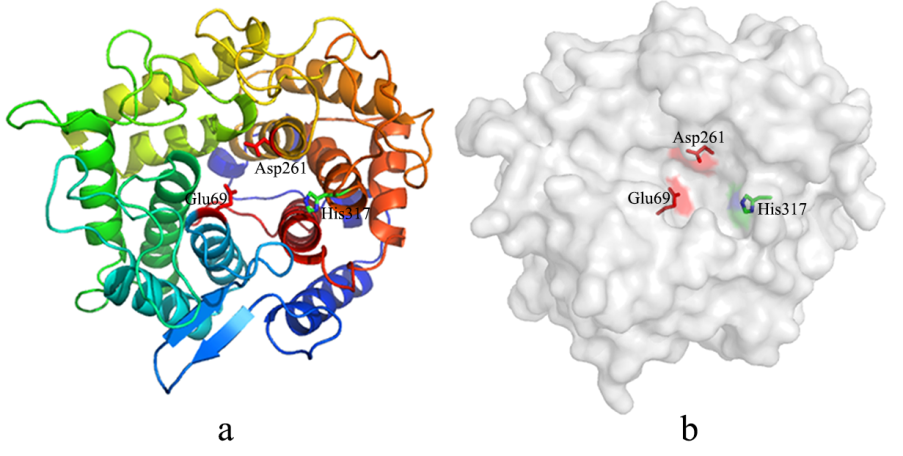
**

**Fig. S3.** Overall structure of PbRex8 (a) Overall structure of PbRex8 in the form of a cartoon. (b) Surface display of PbRex8 structure with three catalytic residues, Glu69, Asp261 and His317. Catalytic residues (Glu69, Asp261) are shown in red sticks. Catalytic residue (His317) contributing to recognition of xylose at the reducing end is shown in green stick.

**
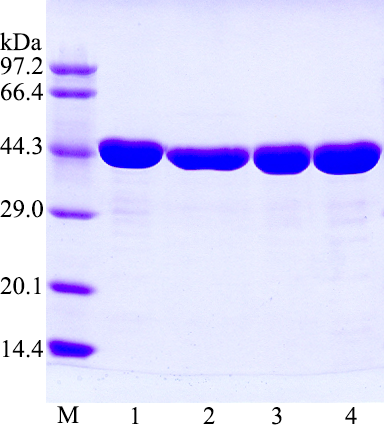
**

**Fig. S4.** SDS-PAGE analysis of the purified PbRex8 and its three mutants. Lane M, low molecular weight protein standards; lane 1, purified mutant R67A; lane 2, purified mutant N122A; lane 3, purified mutant R253A; lane 4, purified PbRex8.

**
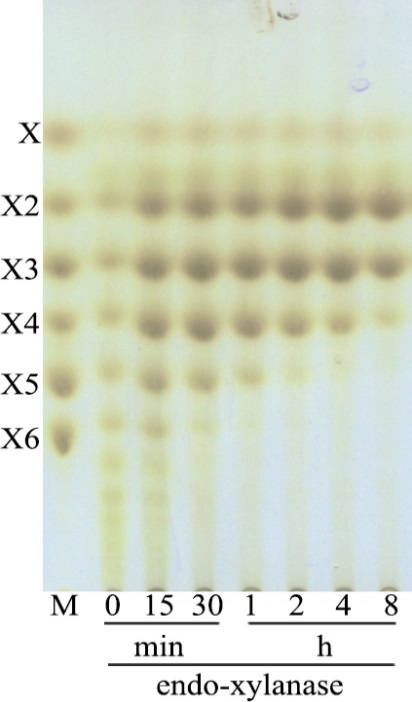
**

**Fig. S5.** TLC analysis of the hydrolysis products of SEMC by PbXyn10A. Incubation times (h or min) are marked. M: marker; X: xylose; X2: xylobiose; X3: xylotriose; X4: xylotetraose; X5: xylopentaose; X6: xylohexaose; endoxylanase: PbXyn10A.


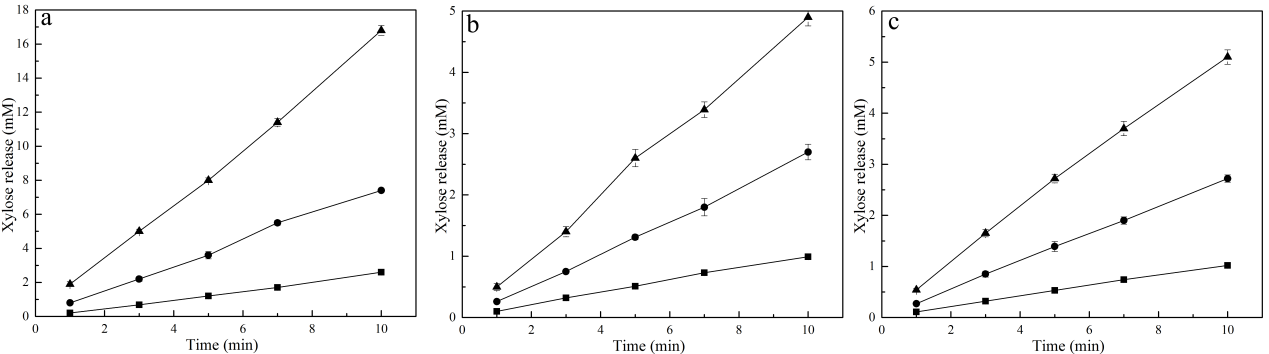


**Fig. S6.** The reaction rates of PbRex8 toward X2 (a), X3 (b) and X4 (c) with different concentrations. a: (■, 35 mM X2 and 40 μg/mL PbRex8), (●, 65 mM X2 and 100 μg/mL PbRex8), (▲, 110 mM X2 and 200 μg/mL PbRex8); b: (■, 7 mM X2 and 5 μg/mL PbRex8), (●, 13 mM X2 and 10 μg/mL PbRex8), (▲, 22 mM X2 and 15 μg/mL PbRex8); c: (■, 5 mM X2 and 5 μg/mL PbRex8), (●, 10 mM X2 and 10 μg/mL PbRex8), (▲, 17.5 mM X2 and 15 μg/mL PbRex8); The determination conditions were at 55 ^o^C and pH 5.5. The values are the average of experiments performed in triplicate.
